# HIF-1α-mediated LAMC1 overexpression is an unfavorable predictor of prognosis for glioma patients: evidence from pan-cancer analysis and validation experiments

**DOI:** 10.1186/s12967-024-05218-3

**Published:** 2024-04-27

**Authors:** Jianrong Bai, Yangyang Zhao, Kaijia Shi, Yonghao Fan, Yanping Ha, Yan Chen, Botao Luo, Yanda Lu, Wei Jie, Zhihua Shen

**Affiliations:** 1 Department of Pathology and Pathophysiology, School of Basic Medicine Sciences, Guangdong Medical University, Zhanjiang, 524023 China; 2 Department of Oncology of the First Affiliated Hospital & Cancer Institute, Hainan Medical University, Haikou, 570102 China; 3 Emergency and Trauma College, Hainan Medical University, Haikou, 571199 China; 4 Department of Pathology, The First Affiliated Hospital of Soochow University, Suzhou, 215000 China

**Keywords:** LAMC1, HIF-1α, Pan-cancer, Gliomas, Prognosis

## Abstract

**Background:**

Laminin subunit gamma-1 (LAMC1) is a major extracellular matrix molecule involved in the tumor microenvironment. Knowledge of the biological features and clinical relevance of LAMC1 in cancers remains limited.

**Methods:**

We conducted comprehensive bioinformatics analysis of *LAMC1* gene expression and clinical relevance in pan-cancer datasets of public databases and validated *LAMC1* expression in glioma tissues and cell lines. The association and regulatory mechanism between hypoxia inducible factor-1α (*HIF-1α*) and *LAMC1* expression were explored.

**Results:**

*LAMC1* expression in most cancers in The Cancer Genome Atlas (TCGA) including glioma was significantly higher than that in normal tissues, which had a poor prognosis and were related to various clinicopathological features. Data from the Chinese Glioma Genome Atlas also showed high expression of *LAMC1* in glioma associated with poor prognoses. In clinical glioma tissues, LAMC1 protein was highly expressed and correlated to poor overall survival. *LAMC1* knockdown in Hs683 glioma cells attenuated cell proliferation, migration, and invasion, while overexpression of *LAMC1* in U251 cells leads to the opposite trend. Most TCGA solid cancers including glioma showed enhancement of *HIF-1α* expression. High *HIF-1α* expression leads to adverse prognosis in gliomas, besides, *HIF-1α* expression was positively related to *LAMC1*. Mechanistically, HIF-1α directly upregulated *LAMC1* promotor activity. Hypoxia (2% O_2_)-treated Hs683 and U251 cells exhibited upregulated HIF-1α and LAMC1 expression, which was significantly attenuated by HIF-1α inhibitor YC-1 and accompanied by attenuated cell proliferation and invasion.

**Conclusions:**

High expression of *LAMC1* in some solid tumors including gliomas suggests a poor prognosis. The hypoxic microenvironment in gliomas activates the HIF-1α/LAMC1 signaling, thereby promoting tumor progression. Targeted intervention on the HIF-1α/LAMC1 signaling attenuates cell growth and invasion, suggesting a new strategy for glioma treatment.

**Supplementary Information:**

The online version contains supplementary material available at 10.1186/s12967-024-05218-3.

## Introduction

Gliomas are common primary cancers in the central nervous system (CNS) of adult males [1]. The World Health Organization (WHO) classification of CNS tumors classifies gliomas into low-grade gliomas (LGGs; WHO I–II) and high-grade gliomas (HGGs; WHO III–IV) by classical histological and molecular characteristics [2]. The survival time after diagnosis of glioma varies among the grades of which glioblastoma multiforme (GBM; WHO grade IV) has the worst overall survival rate, with a 5-year survival rate of < 5% [1]. The clinical behavior of LGG changes and cannot fully predict clinical progression and prognosis in accordance with its histological classification. Some LGG patients show inertia, whereas other patients rapidly progress to GBM. Additionally, differences between observers in histological diagnosis exacerbate uncertainty. Thus, molecular parameters have been used to more accurately classify and predict the prognosis of the various glioma types [3], such as isocitrate dehydrogenase 1 prime 2 (IDH1/2) mutation, ATRX mutation, glioma-CpG island methylation phenotype, O6-methylguanine-DNA methyltransferase methylation, and chromosome arm 1p19q codeletion.

The extracellular matrix (ECM) is the main non-tumor cell microenvironment, providing structural and biochemical support to surrounding cells. It can also be used as a useful biomarker and therapeutic target for tumors [4, 5]. Laminin is a heterotrimer extracellular matrix protein composed of α, β, and γ chains. Five α (α1–α5), four β (β1–β4), and three γ (γ1–γ3) gene forms exist. Various isomers of α, β, and γ chains combine to produce different isotypes of heterotrimeric laminin [6]. Laminin C (LAMC) is the main form of laminin. Three genes have been identified, namely *LAMC1* (formerly known as *LAMB2*), *LAMC2* (also known as *LAMB2T* and *LAMNB2*), and *LAMC3*. LAMC1 is widely expressed in the basement membrane of tissues and is associated with tissue development as well as tumor cell invasion and metastasis, which may contribute to cancer development and progression [7–10]. Glioma cells synthesize, assemble, and secrete various laminin molecules, and laminin subtypes have specific effects on glioma cell migration and invasion in vitro [11]. High expression of laminin has been observed in the tumor parenchyma, tumor invasion front, and tumor vessels of gliomas in some studies, suggesting that laminin is related to the infiltration, migration, and invasion of glioma cells [11–13].

Although some biological functions of *LAMC1* have been reported, the research on *LAMC1* based on pan cancers and the mechanism of regulating *LAMC1* expression are still unclear. This study conducted pan-cancer bioinformatics analysis of the *LAMC1* gene and explored its biological characteristics and mechanism-of-action in glioma. Our results provide new biomarkers and therapeutic targets for clinical treatment of cancers, particularly glioma.

## Methods

### Sample information and differential *LAMC1* expression analysis

*LAMC1* expression data from 31 different tissues were downloaded from the GTEx database (https://commonfund.nih.gov/GTEx). *LAMC1* expression data from tumor cell lines were downloaded from the CCLE database (https://portals.broadinstitute.org/ccle/). GTEx and CCLE database analyses employed the Sangerbox analysis tool (http://www.sangerbox.com/tool). RNA sequencing, and related clinical and phenotypic data were downloaded from TCGA, including 11,069 samples from 33 cancers, using UCSC Xena (https://xena.ucsc.edu/), an online tool used to explore gene expression. Strawberry Perl (Version 5.32.0, http://strawberryperl.com/) was used to extract *LAMC1* gene expression data from these downloaded datasets and draw them in a data matrix for subsequent analysis. Using the downloaded data and comparing expression levels between cancer and matched normal samples, *LAMC1* expression in 31 normal tissues and 21 tumor cell lines was evaluated. All gene expression data were standardized by log2 conversion, and two sets of t-tests were carried out for the tumor types. *P* < 0.05 was considered to indicate differential expression between tumor and normal tissues. R software (version 4.0.4, https://www.R-project.org) was used to analyze the data, and the R software package ggpubr was used to draw a box diagram. For some tumors without normal or highly restricted normal tissue, we used the expression analysis-box map module in GEPIA2 (http://gepia2.cancer-pku.cn/#index/) to obtain the box diagram of expression differences between tumor and corresponding normal tissues in the GTEx database.

### Prognosis and clinicopathological correlation analyses based on *LAMC1* expression levels in pan-cancer data from TCGA database

Survival and clinical phenotype data were extracted for each sample downloaded from TCGA. Overall survival (OS), disease-specific survival (DSS), disease-free interval (DFI), and progression-free interval (PFI) were selected to examine the relationship between *LAMC1* expression and patient prognosis. Survival analyses of each cancer type were performed using the Kaplan–Meier method and log-rank test. A median *LAMC1* expression level was chosen as the cutoff value for the human cancer dichotomy, thereby dividing respective patients into high- and low-risk groups. Survival curves were drawn using R packages survival and survminer. Cox analysis was conducted using R packages survival and forestplot to explore the relationship between *LAMC1* expression and pan-cancer prognoses. Two clinical phenotypes, including patient age and tumor stage, were selected and their relationship with *LAMC1* expression was investigated. Clinical phenotype correlation analyses were conducted using R packages limma and ggpubr. *P* < 0.05 was considered significant.

### Clinicopathological analysis of *LAMC1* expression in Chinese glioma patients in the CGGA database

Chinese Glioma Genome Atlas (CGGA) datasets (http://www.cgga.org.cn/) were used to re-assess the expression and prognostic significance of *LAMC1*. Two datasets (DataSet IDs: mRNAseq_693 and mRNAseq_325) containing 693 and 325 samples of LGG and GBM, respectively, were downloaded and analyzed. Data were corrected in batches and integrated using R packages limma and sva. We used survival and survminer packages to plot a survival curve for *LAMC1* expression and glioma. Univariate and multivariate Cox analyses were used to determine and evaluate independent prognostic indicators of OS. Receiver operating characteristic (ROC) curves of *LAMC1* and glioma at 1, 3, and 5 years were plotted using the K-M method in the survival ROC package. The correlations between *LAMC1* expression and clinical characteristics were plotted using beeswarm in the R package.

### Cell culture and *LAMC1* gene targeted intervention

Human representative glioma cell lines U87MG, U251, A172, and Hs683 were obtained from Genechem (Shanghai, China). Cells were cultured in DMEM containing 10% fetal bovine serum (Thermofisher, Shanghai, China) at 37 °C in a humidified atmosphere with 5% CO_2_. Among the cell lines, Hs683 cells were confirmed high expression while U251 showed low expression of *LAMC1* by western blotting (Additional file 1: Fig. S1A). For *LAMC1* knockdown in Hs683 cells, a lentivirus carrying shRNA-#3 against the human *LAMC1* target sequence 5ʹ-CAAAGTTCTCAAGTCCTAT-3ʹ was constructed by Genechem. For overexpression of *LAMC1* in U251 cells, a lentivirus carrying protein-coding region for human *LAMC1* gene was designed and packaged by Genechem. After viral infection, cell lines were screened using puromycin stress and further validated by western blotting of LAMC1 expression (Additional file 1: Fig. S1B–E).

### Tissue microarray and immunohistochemistry (IHC)

A tissue microarray of glioma (Cat: HBraG159Su01) and normal brain tissue sections were purchased from Shanghai Outdo Biotech (Shanghai, China). IHC was performed using a DAKO automatic IHC instrument in accordance with the manufacturer’s protocol. The array was incubated with an anti-LAMC1 antibody (Cat#: ABP55085, Abbkine, Wuhan, China) at a 1:100 dilution overnight at 4 °C and developed using Dako Liquid DAB. The scoring system of the staining intensity was as follows: negative (0 points); weak (1 points); moderate (2 points); strong (3 points). The positive range percentage criteria were as follows: 0%–25% positive cells (1 points); 26%–50% positive cells (2 points); 51%–75% positive cells (3 points); 76%–100% positive cells (4 points). Stained samples were analyzed under microscope (Aperio XT, LEICA, Germany). The total immunoreactive score was evaluated independently by two pathologists by summing by the nuclear and cytoplasm/membrane staining scores. For cases where the scores of two pathologists are inconsistent, a re-evaluation score should be made to reach a consensus.

### Cell counting kit-8 (CCK-8) and colony formation assays

Cells were seeded in a 96-well plate (2 × 10^3^ cells/well) and incubated for 0, 24, 48, 72, 96, and 120 h at 37 °C with 5% CO_2_. Then, 10 µl CCK-8 solution (Beyotime, Nantong, China) was added to each well at 2 h before the incubation time ended. Against a background control, sample absorbance was measured at 450 nm with a microplate reader. Each group was repeated 5–6 wells. For the colony formation assay, cells were seeded in 6-well plates at 1500 cells/well and cultured for 12 days. Colonies were fixed for 30 min with 4% paraformaldehyde and stained for 15 min with 0.1% crystal violet. The number of colonies (≥ 50 cells) was counted manually under an inverted microscope. Each group was assessed in three wells.

### Cell migration and invasion assays

Cell migration and invasion were evaluated by transwell assays and wound-healing assays as described previously [14]. Transwell plates (Corning, NY, USA) with 8 μm pore-sized polycarbonate filters were used for cell migration assays. Chambers coated with Matrigel were used to evaluate cell invasion. Each group was assessed in at least three wells.

### Hypoxic treatment of glioma cells

A three-gas incubator was used for cell culture under hypoxic conditions as described previously [15]. Hs683 and U251 cells were cultured under normoxic (21% O_2_ and 5% CO_2_) or hypoxic (2% O_2_, 5% CO_2_, and 93% N_2_) conditions for various times. Total proteins were isolated for relevant experiments. To inhibit HIF-1α expression, 10 μmol/L lificiguat (YC-1; MCE, Shanghai, China) was used.

### Western blotting

Cellular proteins were extracted using RIPA buffer (Beyotime). Then, 50 μg of proteins was subjected to 10% SDS-PAGE. PVDF membranes were probed with antibodies against LAMC1 (Cat#: 6776-1-Ig, 1:200 dilution; Proteintech, Wuhan, China), HIF-1α (Cat#: ab179483, 1:1000 dilution; Abcam, Cambridge, UK), or β-tubulin (Cat#: ab179513, 1:1000 dilution; Abcam). After developing the membrane with ECL reagent, bands were imaged using a gel imaging system (Tanon, Shanghai, China).

### Dual luciferase reporter assay

Potential HIF-1α-binding sites in the *LAMC1* promoter region were predicted using the JASPAR database (http://jaspar.genereg.net/) [16]. pGL4-based luciferase reporter plasmids containing wildtype and overexpressed *LAMC1* promoters, a *HIF-1α* (NM_001530) overexpression plasmid, and Renilla internal reference plasmid pRL-TK were constructed by Genechem. The plasmids were co-transfected into 293 T cells Genechem), and luciferase activity was detected by the Dual-Luciferase Reporter Assay System (Cat#: E1910, Promega China, Beijing) at 48 h after transfection. Firefly and Renilla luminescence in sample wells was detected by a microplate reader. The Firefly/Renilla luminescence value represented luciferase activity. Each group was analyzed in three wells.

### Statistical analysis

*LAMC1* and *HIF-1α* expression levels were analyzed using the non-parametric Mann–Whitney or Kruskal–Wallis test. The Kaplan–Meier method and log-rank test were used to evaluate the association between *LAMC1* and *HIF-1α* expression and prognosis. Cox regression analysis was used to identify factors that may have a significant influence on survival. We used the GEPIA2 database to analyze the correlation between *LAMC1* and *HIF-1α* expression. The immunoreactive scores of LAMC1 protein in tissue arrays were calculated using the Wilcoxon signed-rank test. The chi-squared test was performed to analyze the association between the LAMC1 protein level and clinicopathological parameters. Data from in vitro experiments are presented as mean ± SD. The unpaired t-test or one-way ANOVA was used accordingly. Statistical analyses were carried out by GraphPad Prism 8 and R version 4.0.2 software. *P* < 0.05 was considered to indicate a statistically significant difference.

## Results

### *LAMC1* expression in human pan-cancers

**Fig. 1:**
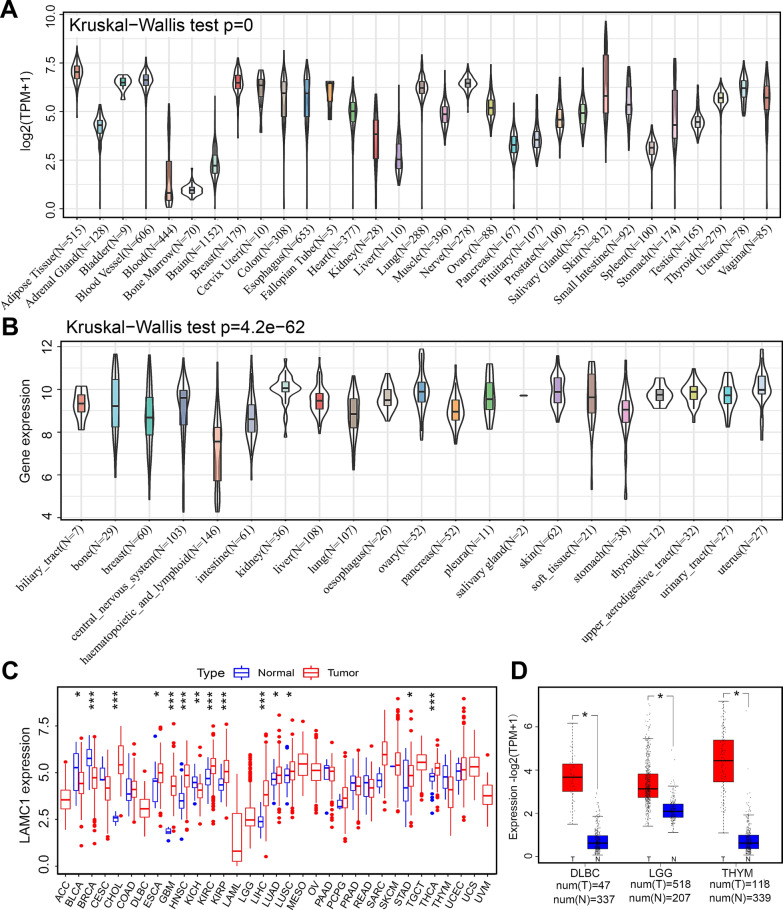
Differential expression of the *LAMC1* gene in pan-cancers. **A** *LAMC1* expression in normal human tissues. **B** *LAMC1* expression in human tumor cell lines. **C** Comparison of *LAMC1* expression between tumor and normal samples from TCGA database. *P < 0.05, **P < 0.01, ***P < 0.001. **D** Integrated GTEX and TCGA databases to analyze *LAMC1* expression between tumor and normal samples of DLBC, LGG, and THYM. *P < 0.05

GTEx datasets showed various levels of *LAMC1* gene expression in humans and a low expression level in brain tissues (Fig. 1A). However, a relatively high expression level of *LAMC1* was found in CNS tumors (Fig. 1B). In TCGA data, *LAMC1* was highly expressed in CHOL, ESCA, GBM, HNSC, KIRC, KIRP, LIHC, LUAD, LUSC, STAD, and THCA. Conversely, *LAMC1* expression was downregulated in tumors in BLCA, BRCA, and KICH relative to normal tissue (Fig. 1C). After adding normal tissues from the GTEx dataset as controls, we found enhanced expression of *LAMC1* in DLBC, LGG, and THYM (Fig. 1D).

### Multifaceted prognostic features of *LAMC1* across pan-cancers

**Fig. 2:**
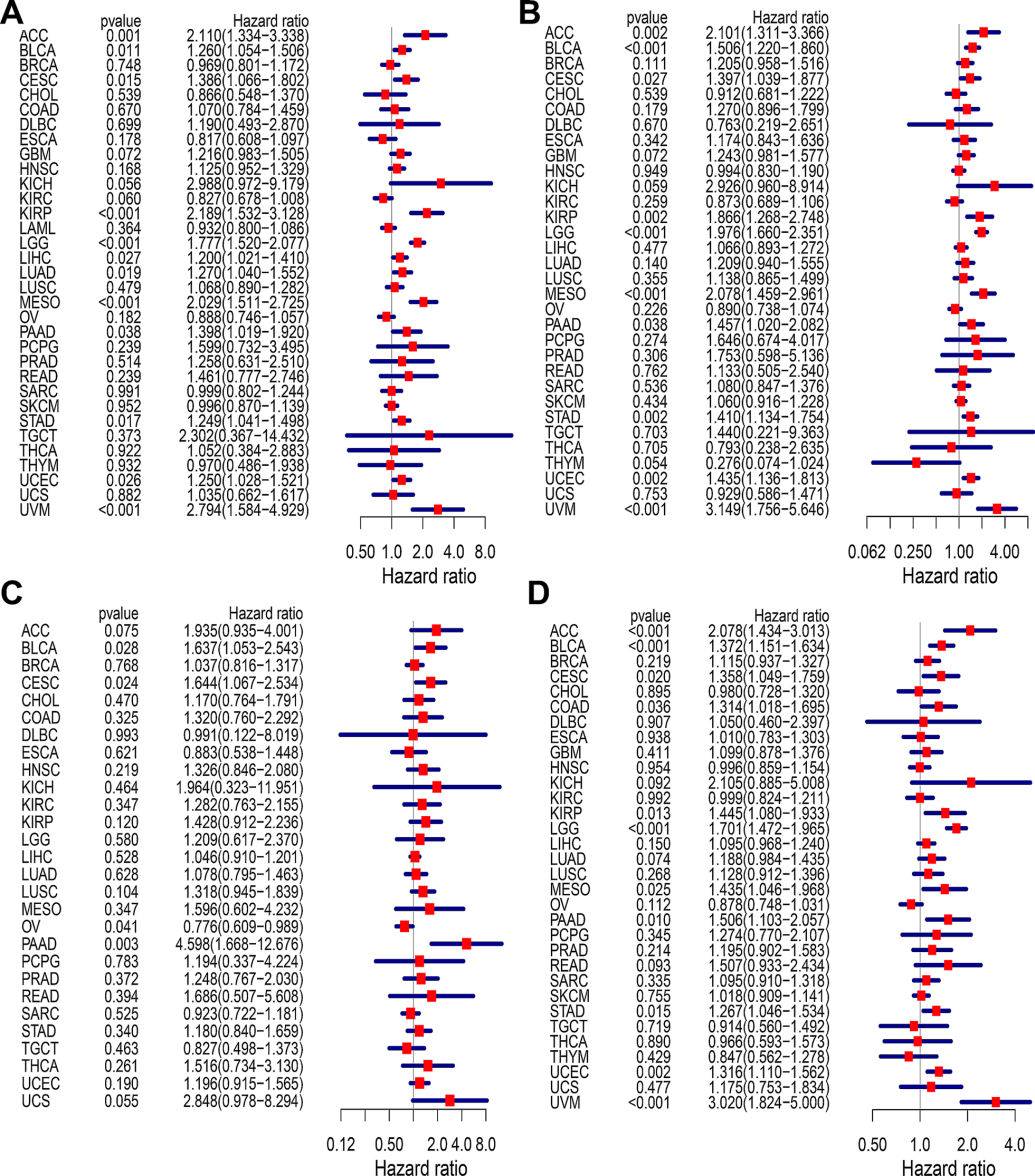
Association between *LAMC1* expression and OS, DSS, DFI, and PFI in pan-cancers. **A** Forest plot of the association of *LAMC1* expression with OS in TCGA tumors. **B** Forest plot of the association of *LAMC1* expression with DSS in TCGA tumors. **C** Forest plot of the association of *LAMC1* expression with DFI in TCGA tumors. **D** Forest plot of the association of *LAMC1* expression with PFI in TCGA tumors

**Fig. 3:**
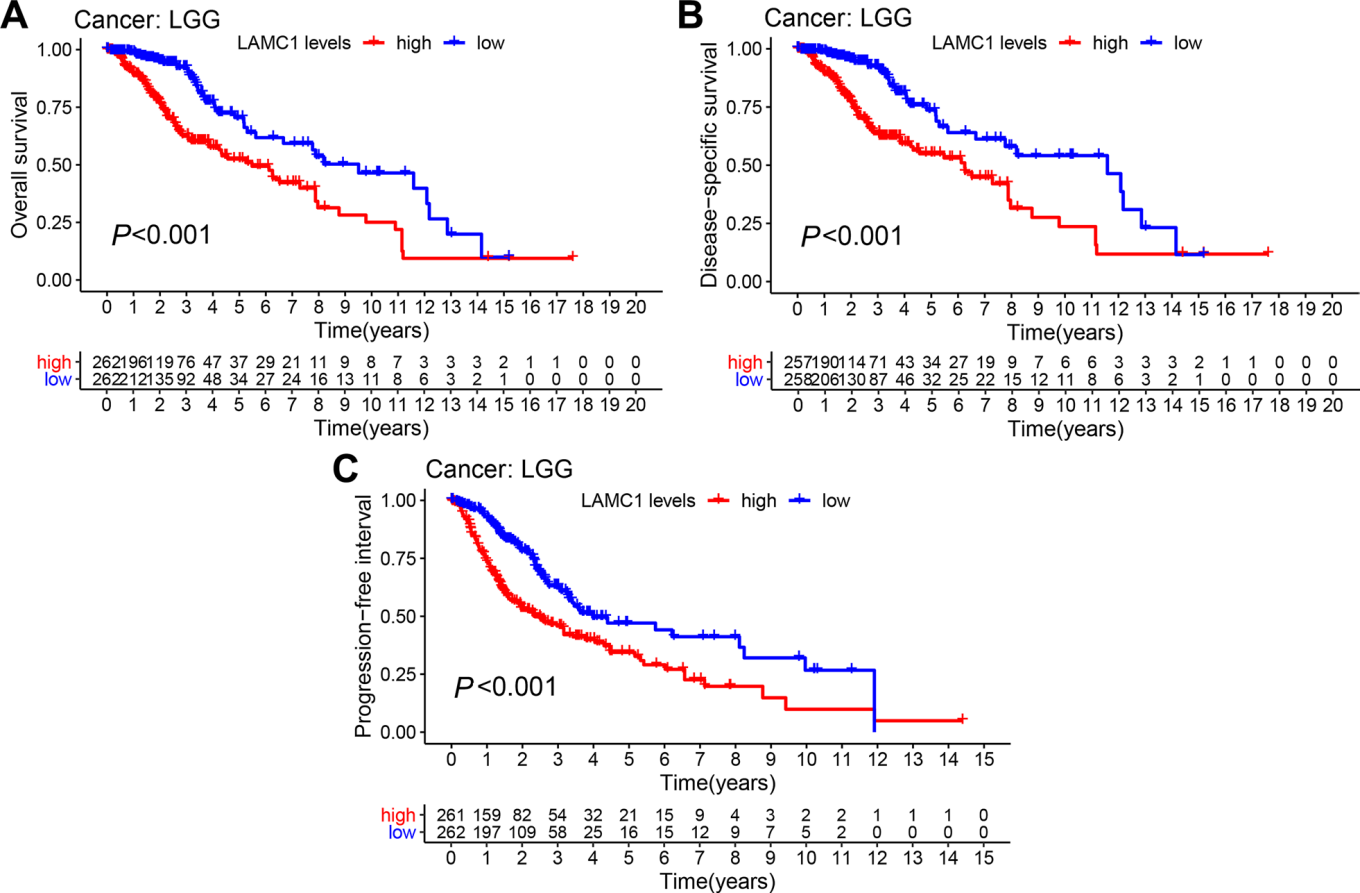
Kaplan–Meier analysis of the association between *LAMC1* expression and OS, DSS, and PFI of LGG patients. **A** Association between *LAMC1* expression and OS of LGG patients. **B** Association between *LAMC1* expression and DSS of LGG patients. **C** Association between *LAMC1* expression and PFI of LGG patients

Cox proportional risk model analysis showed that *LAMC1* expression levels were significantly correlated to OS of patients with KIRP, LGG, MESO, and UVM (*P* < 0.001) and many other cancer types (*P* < 0.05) (Fig. 2A). *LAMC1* expression was associated with DSS of various cancers, including BLCA, LGG, MESO, and UVM (*P* < 0.001) (Fig. 2B). High *LAMC1* expression was correlated to DFI in patients with BLCA, CESC, OV, and PAAD (*P* < 0.05) (Fig. 2C). In terms of the association between high *LAMC1* expression levels and PFI, a forest map showed a poor prognosis of patients with ACC, BLCA, LGG, and UVM (*P* < 0.001) (Fig. 2D). Kaplan–Meier survival analysis also showed that LGG patients with high *LAMC1* levels had poor OS, DSS, and PFI (*P* < 0.001, Fig. 3).

### Clinicopathological analysis of *LAMC1* expression in Chinese glioma patients

**Fig. 4:**
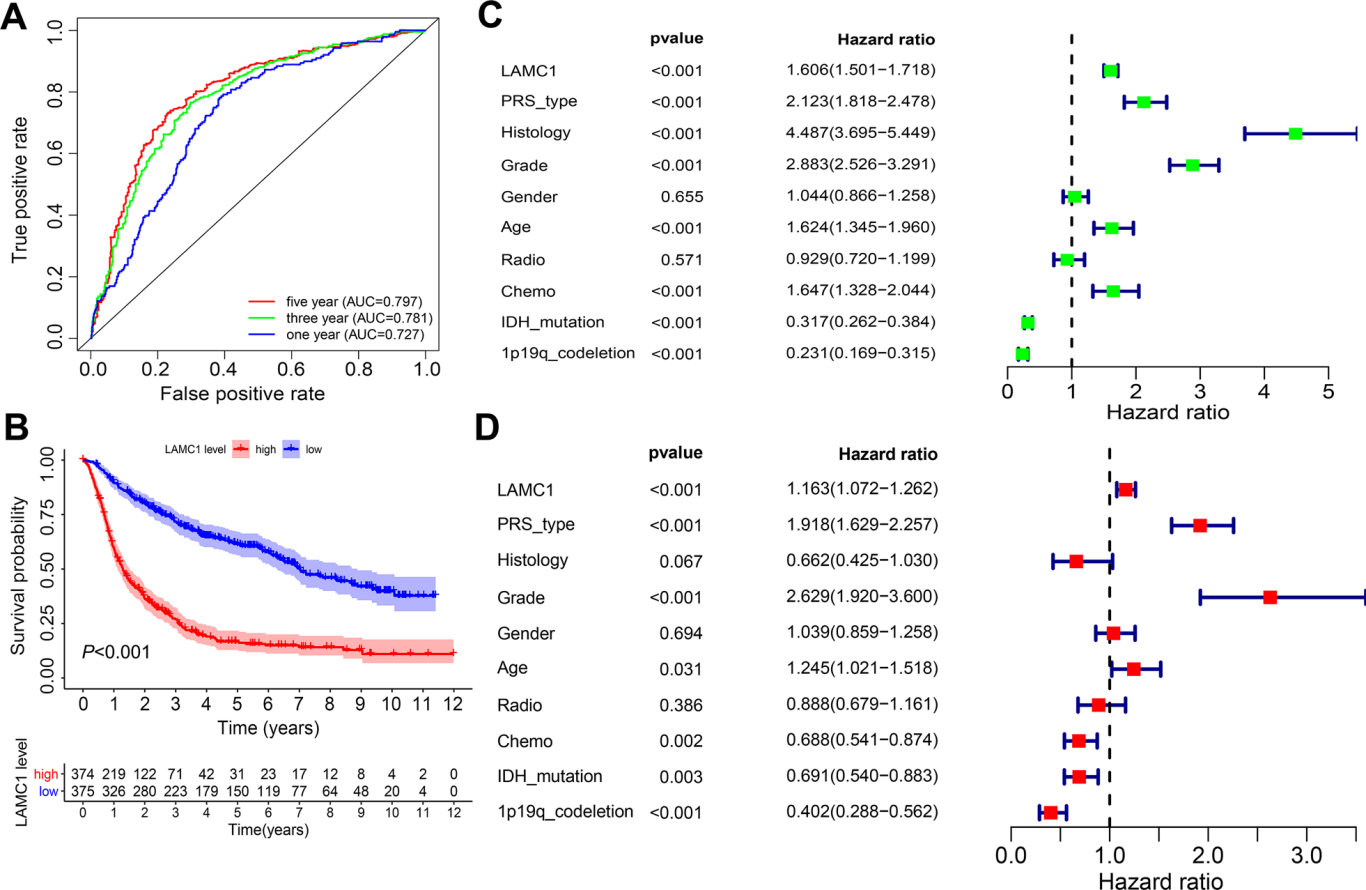
Clinicopathological analysis of *LAMC1* expression in the CGGA database. **A** Receiver operator characteristic (ROC) curve of *LAMC1* expression. AUC: area under the curve. **B** Survival analysis of glioma patients in high and low *LAMC1* expression groups. *P* < 0.001. **C** Univariate analysis of *LAMC1* expression. **D** Multivariate analysis of *LAMC1* expression

Bioinformatics analysis of *LAMC1* expression in gliomas was performed using the CGGA database [17]. ROC curve analysis showed that *LAMC1* expression was a predictor of 1-year (AUC = 0.727), 3-year (AUC = 0.781), and 5-year (AUC = 0.797) survival with a good predictive value (Fig. 4A). Kaplan–Meier survival analysis showed that high *LAMC1* expression was associated with a poor prognosis of patients with glioma (Fig. 4B). Univariate Cox analysis showed that *LAMC1* expression, primary-recurrent-secondary (PRS) type, histology, grade, age, and chemotherapy were high-risk factors (HR > 1), and IDH mutation and 1p19q codeletion were low-risk factors (HR < 1) (Fig. 4C). Multivariate Cox analysis showed that *LAMC1* expression (*P* < 0.001, HR = 1.163), PRS type (*P* < 0.001, HR = 1.918), grade (*P* < 0.001, HR = 2.619), IDH mutation (*P* = 0.003, HR = 0.691) and 1p19q codeletion (*P* < 0.001, HR = 0.402) may also be independent prognostic factors (Fig. 4D).

### Correlation between *LAMC1* expression and clinical phenotypes of various cancers

**Fig. 5:**
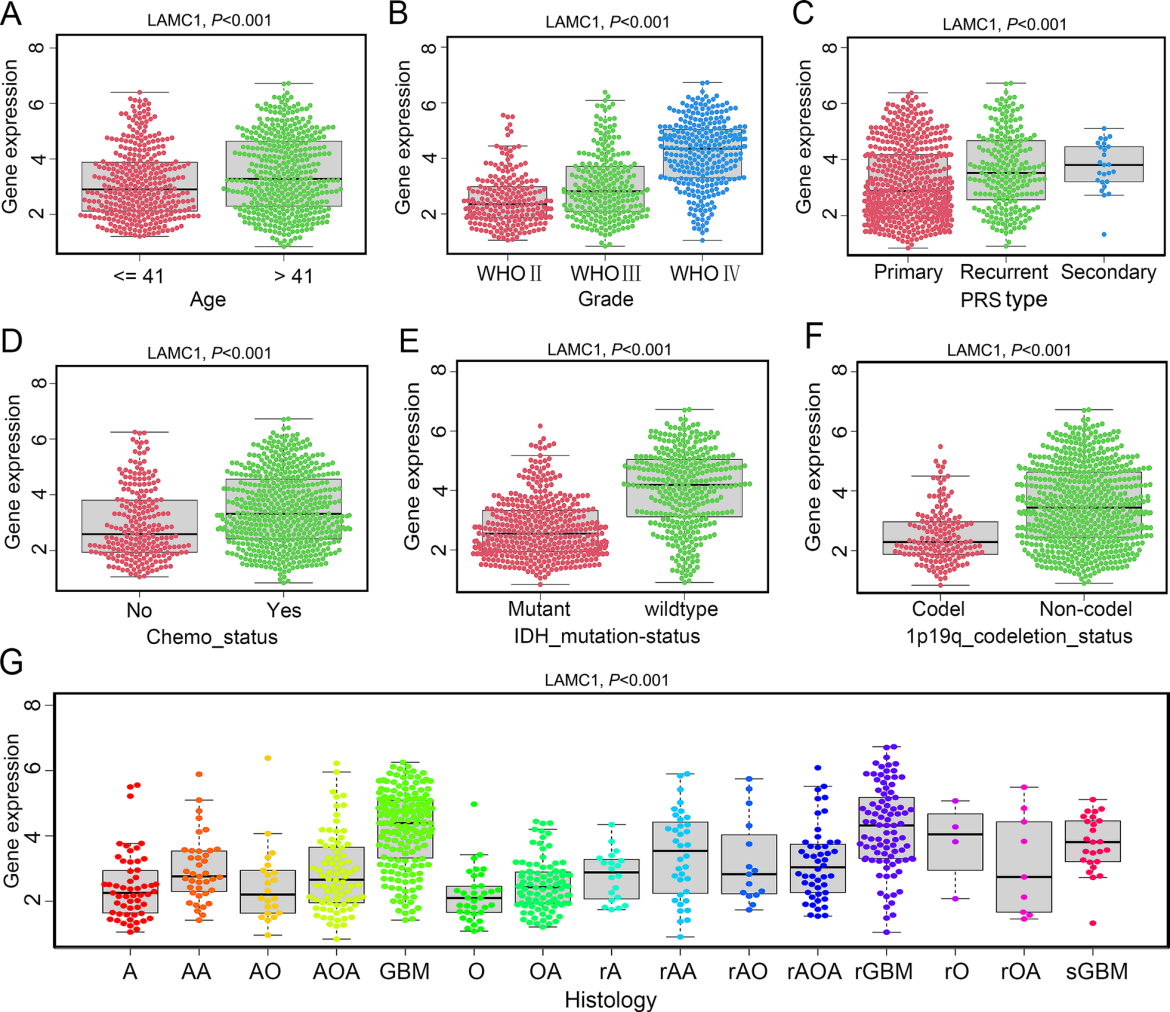
Correlation of *LAMC1* expression levels with clinical features in glioma patients in the CGGA database. **A** Age. **B** Grade. **C** PRS type. **D** Chemotherapy. **E** IDH mutation. **F** 1p19q codeletion. **G** Histology

Next, we examined differential expression of *LAMC1* in patients with each tumor type in accordance with age and the tumor stage in TCGA database. We found that patients older than 65 years of age with BRCA (*P* = 0.00032), COAD (*P* = 0.007), KIRC (*P* = 0.011), LIHC (*P* = 0.0078), or PAAD (*P* = 0.0018) had low *LAMC1* expression levels. *LAMC1* expression was higher in THYM (*P* = 0.012) and UCEC (*P* = 0.041) patients over 65 years of age (Additional file 1: Fig. S2A). We also found statistical significance between *LAMC1* expression and the partial tumor stage in eight cancer types, including BLCA, COAD, HNSC, KICH, KIRC, LUSC, PAAD, and UVM. Notably, *LAMC1* expression increased with the stage in most tumors, suggesting that high *LAMC1* expression is associated with tumor progression (Additional file 1: Fig. S2B). Clinicopathological characteristic correlation analysis of the CGGA data demonstrated that the expression level of *LAMC1* was remarkably related to age (Fig. 5A), grade (Fig. 5B), PRS type (Fig. 5C), chemotherapy (Fig. 5D), and histology (Fig. 5G) in glioma samples. Recently, it has been widely recognized that IDH mutations and 1p19q codeletion suggest a favorable prognosis of gliomas [18]. In this study, high expression levels of *LAMC1* were found in IDH-wildtype and 1p19q non-coding glioma compared with IDH-mutants or 1p19q codeletions (Fig. 5E, F). These findings suggest that *LAMC1* participates in the clinical development of glioma.

### Correlation of *LAMC1* protein expression to clinicopathological parameters and prognosis in the glioma cohort

**Fig. 6:**
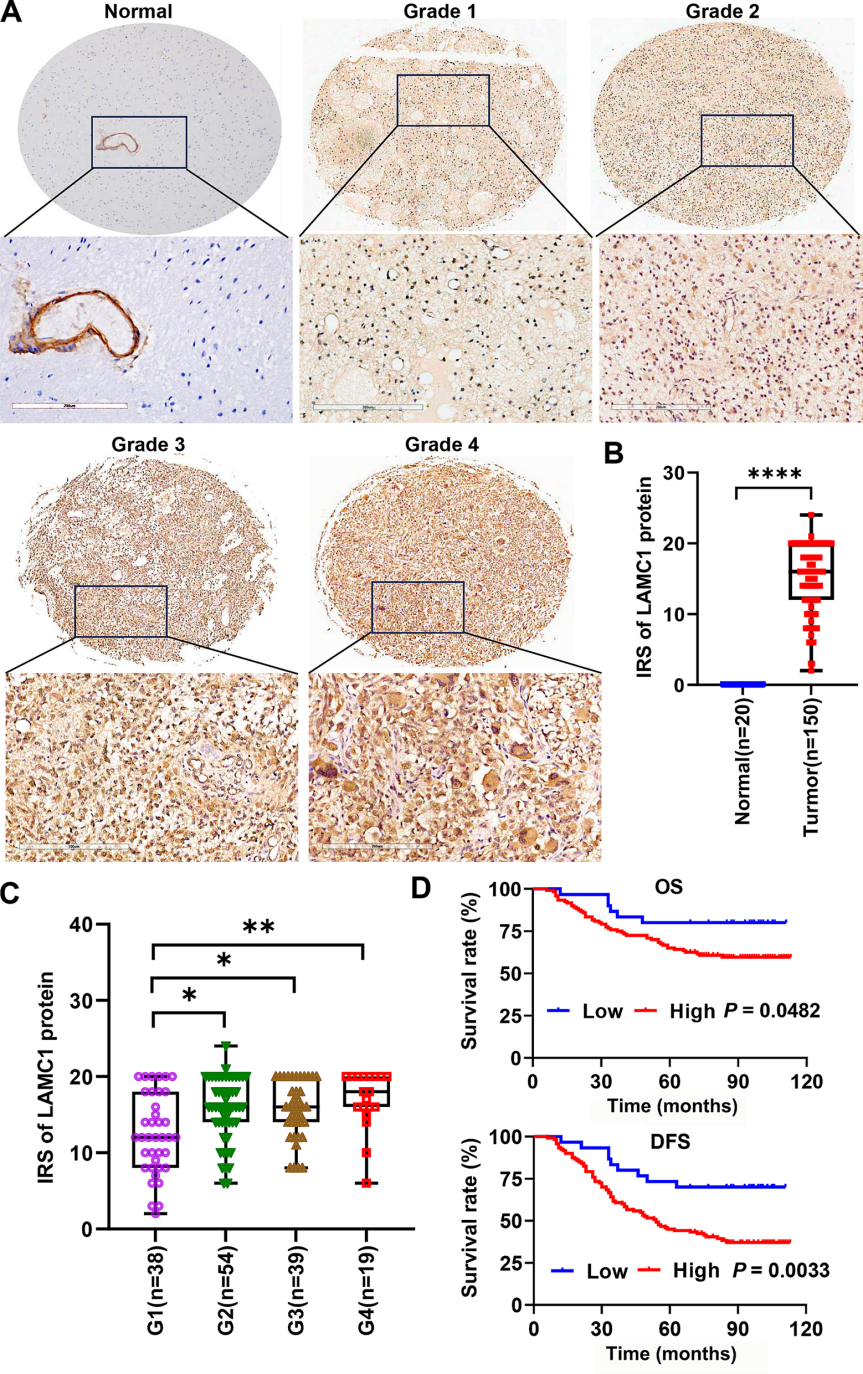
LAMC1 protein expression correlates to clinicopathological parameters of glioma patients based on tissue array analysis. **A** Representative images of the IHC staining intensity of LAMC1 in gliomas with different pathological grades and normal brain tissue. Bars = 200 μm. **B** Immune response score (IRS) of LAMC1 in gliomas and normal controls, ****P < 0.0001. **C** IRS of LAMC1 protein expression in glioma tissues with different grades. *P < 0.05, **P < 0.01. **D** Kaplan–Meier analysis of OS and DFS of glioma patients in accordance with LAMC1 protein expression levels

We explored the correlation between LAMC1 protein expression and clinicopathological parameters based on the information of tissue microarray cases. The positive sites for LAMC1 protein expression in the tissue were both cytoplasmic and nuclei. The calculated total immunoreactive scores of LAMC1 expression ranged from 2 to 24 in all samples. We divided the cases into subgroups of high (IHC score > 10) and low (IHC score ≤ 10) LAMC1 expression based on the cutoff point determined by X-tile software related to survival time and status. LAMC1 expression was correlated to the degree of pathological grade and postoperative recurrence of patients (*P* < 0.05). Additionally, no association was found between LAMC1 expression and gender (*P* = 0.798) or age (*P* = 0.314) (Additional file 1: Table S1). Representative images of the IHC staining intensity of LAMC1 in gliomas with different pathological grades and normal brain tissue are shown in Fig. 6. LAMC1 was nearly negative in normal brain tissue (Fig. 6A, B). As shown in Fig. 6C, glioma patients with higher pathological grades (G2–4) had higher total immunoreactive scores for LAMC1 than those with a lower grade (G1) (*P* < 0.05). Survival analysis by the Kaplan–Meier method with the log-rank test indicated that patients with a high level of LAMC1 had worse outcomes and shorter OS (*P* = 0.0482) and DFS (*P* = 0.0033) than those with low LAMC1 expression (Fig. 6D). Age and pathological grade were independent prognostic indicators in both univariate and multivariate Cox analysis models (Additional file 1: Table S2).

### *LAMC1* knockdown inhibits while overexpression promotes glioma cell proliferation, migration, and invasion

**Fig. 7:**
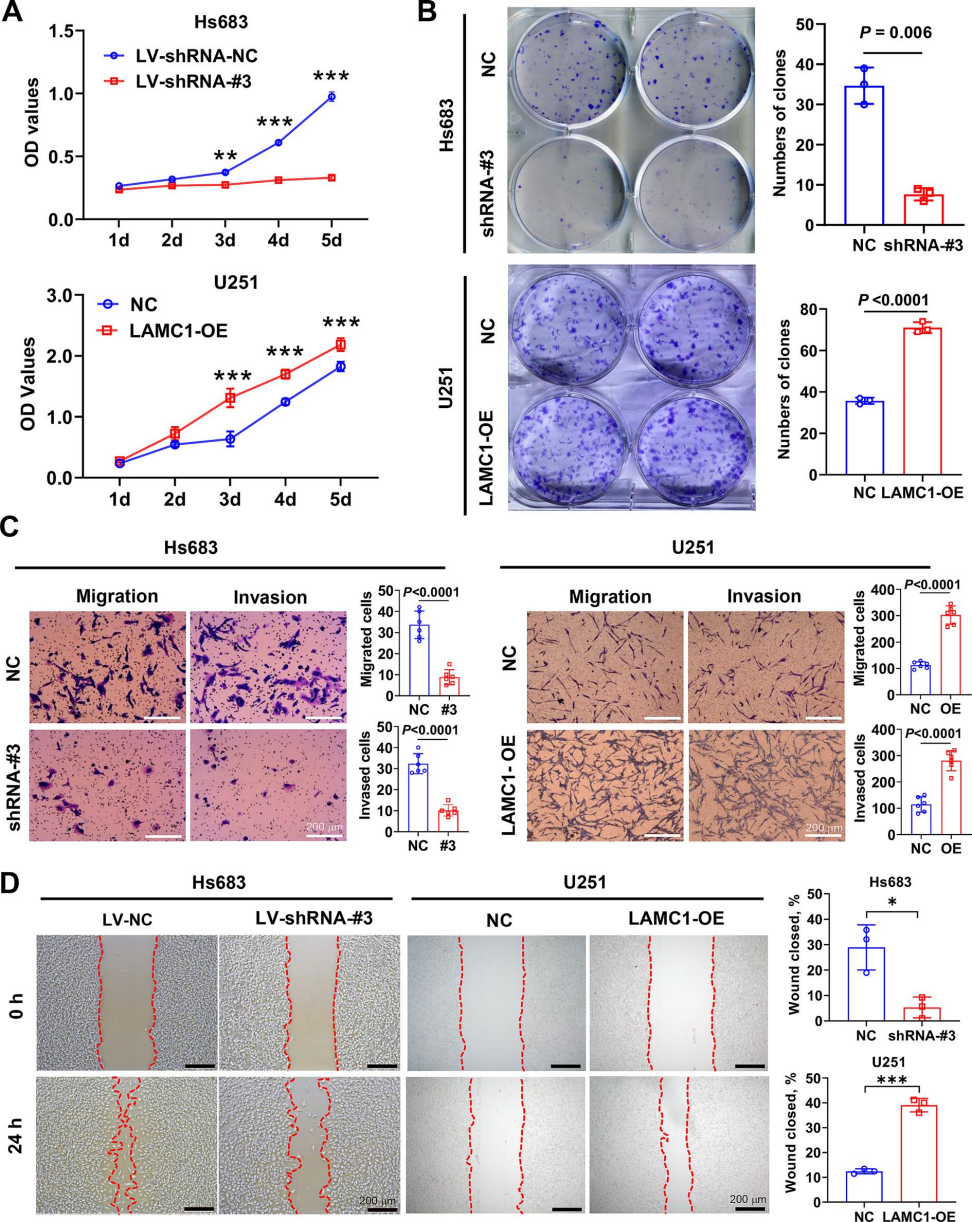
Effects of *LAMC1* intervention on proliferation, migration, and invasion of glioma cells. **A** Viability of glioma cells assessed by CCK-8 assays, ** *P*<0.01, ***P < 0.001. **B** Proliferation ability glioma cells assessed by colony formation assays. **C** Migration and invasion abilities of glioma cells were compared by transwell assays. **D** Migration and repair abilities of glioma cells were compared by wound-healing assay. *P < 0.05, ***P < 0.001

Hs683 cells with highest *LAMC1* expression and U251 cells with lowest *LAMC1* expression were chosen for subsequent experiments. Fluorescence microscopy showed that lentiviral particles had a higher infection efficiency in glioma cells, and western blotting verified the efficacy in *LAMC1* knockdown or overexpression (Additional file 1: Fig. S1). CCK-8 assays showed that cell proliferation in the *LAMC1* knockdown group was significantly slower than that in the control group, and *LAMC1* overexpression was significantly enhanced the viability compared with the relevant controls (*P* < 0.001, Fig. 7A), the results of colony formation were consistent with the CCK-8 findings (Fig. 7B). Furthermore, *LAMC1* knockdown decreased while *LAMC1* overexpression increased the number of cells that migrated or invaded through transwell chambers (*P* < 0.001, Fig. 7C). Wound-healing assay results were consistent with the migration assay results (Fig. 7D). These data collectively indicate that *LAMC1* promotes glioma cell proliferation, migration, and invasion.

### High *HIF-1α *expression promotes clinical progression and correlates positively to *LAMC1* expression in gliomas

**Fig. 8:**
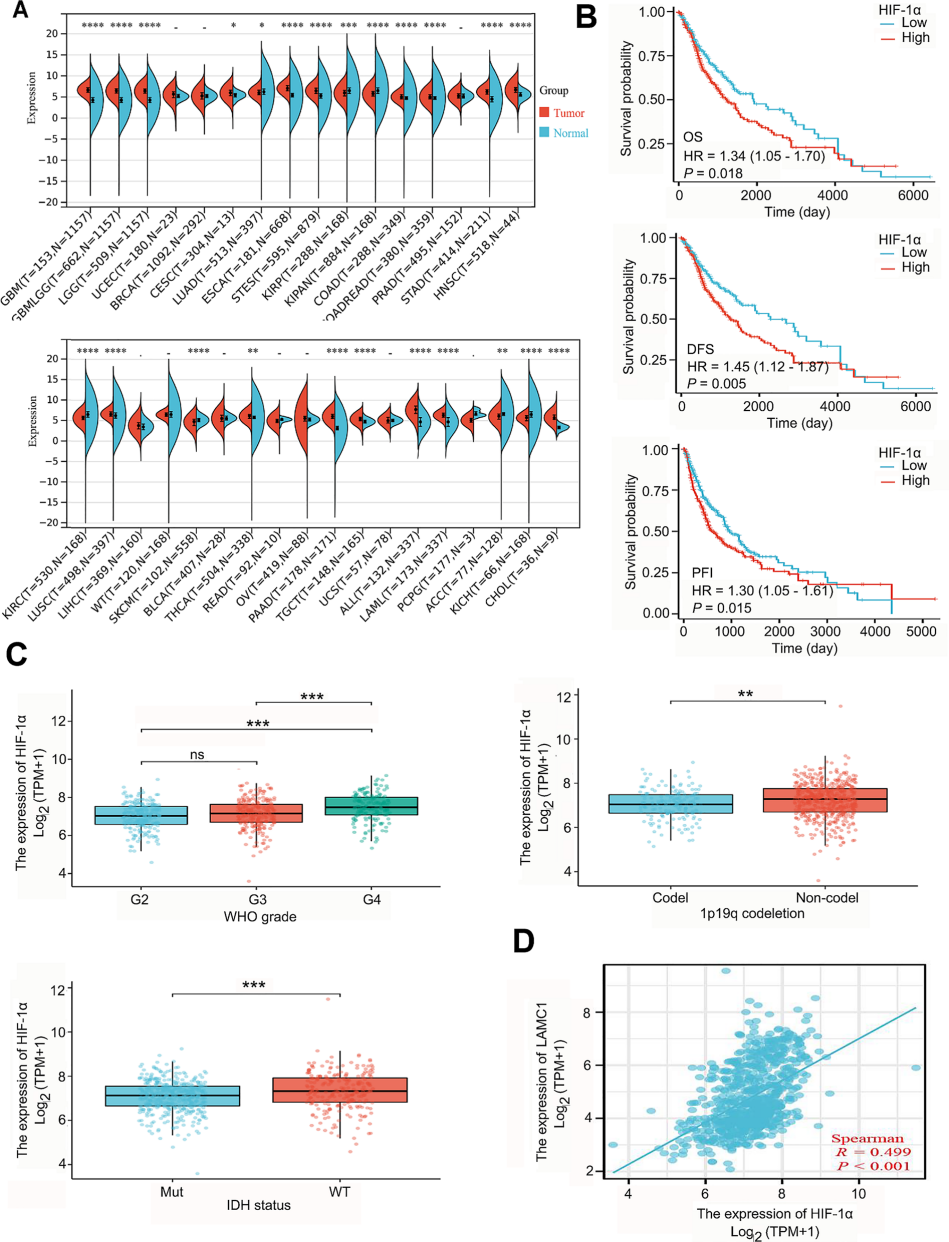
Expression of *HIF-1α* in TCGA pan-cancers and its prognosis, clinical pathology and correlation with *LAMC1* in gliomas. **A** Expression of *HIF-1α *in pan-cancer and control tissues. *P < 0.05, **P < 0.01, ***P < 0.001, ****P < 0.0001. **B** The relationship between *HIF-1α* and the OS, DFS and PFI of glioma. **C** Relationship between *HIF-1α* expression and the pathological grade, 1p19q codeletion and IDH mutation of glioma patients. **D** Correlation between *HIF-1α* and *LAMC1* expression in glioma

*HIF-1α* expression was significantly upregulated in various TGCA solid tumors, including GBM, GBMLGG, LGG, CESC, ESCA, STES, COAD, STAD, HNSC, LUSC, THCA, PAAD, TGCT, ALL, LAML, and CHOL. Significant downregulation of *HIF-1α* expression was also observed in KIRP, KIPAN, KIRC, SKCM, ACC, and KICH (Fig. 8A). Moreover, high *HIF-1α* expression associates with poor prognosis of gliomas (Fig. 8B), as well as the advanced pathological grade, 1p19q codeletion and IDH mutation status (Fig. 8C). Based on clinical glioma tissue, we detected that the expression level of HIF-1α protein increases with the increase of pathological grade (Additional file 1: Fig. S3). Correlation analysis showed that *HIF-1α* expression in normal brain tissue and glioma was positively correlated to *LAMC1* (*r* = 0.499, *P* < 0.001; Fig. 8D).

### Effects of hypoxia on *LAMC1* expression in glioma

**Fig. 9:**
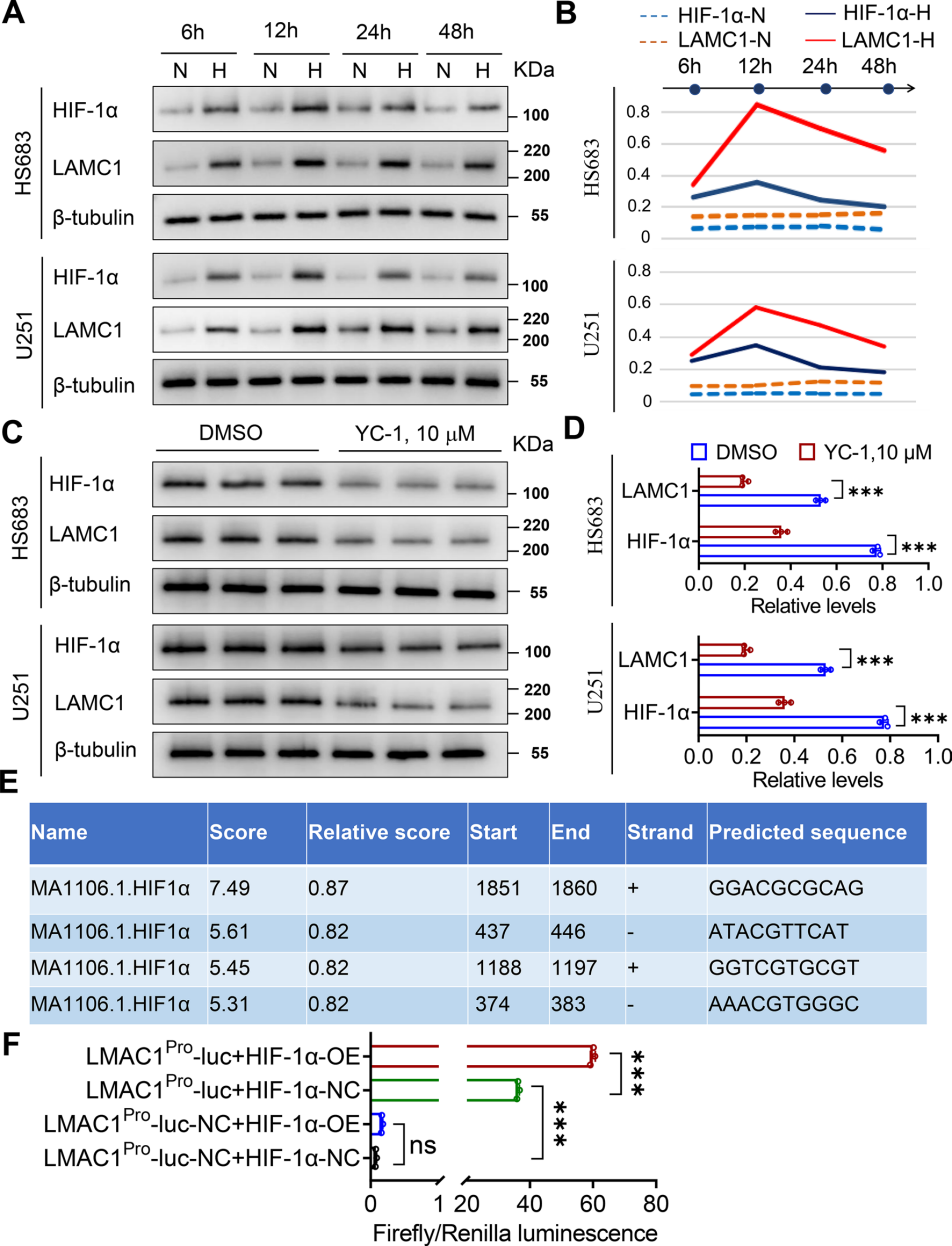
HIF-1α regulates LAMC1 expression in glioma cells. **A, B** Western blot and semi-quantitative analysis of LAMC1 and HIF-1α protein expression in glioma cells under normoxia (N) and hypoxia (H). **C, D** Western blot and semi-quantitative analyses of HIF-1α inhibitor YC-1-induced suppression of LAMC1 and HIF-1α protein expression in glioma cells at 12 h of hypoxia. ***P < 0.001. **E** Prediction of binding sites between the *LAMC1* promoter and HIF-1α protein. **F** Results of luciferase reporter assays. ***P < 0.001; ns, no statistical difference. LAMC1^Pro^-luc-HIF-1α-OE, LAMC1 promoter plus HIF-1α overexpression; LAMC1^Pro^-luc-HIF-1α-NC, LAMC1 promoter plus negative control of HIF-1α; LAMC1^Pro^-luc-NC-HIF-1α-OE, negative control of LAMC1 promoter plus HIF-1α overexpression; LAMC1^Pro^-luc-NC-HIF-1α-NC, negative control of LAMC1 promoter plus negative control of HIF-1α

LAMC1 and HIF-1α expression varied with the change in hypoxic exposure time (Fig. 9A, B, Additional file 1: Fig. S4A). After hypoxic treatment for 12 h, LAMC1 and HIF-1α protein expression was the highest. Notably, while confirming that the HIF-1α inhibitor YC-1 has no significant cytotoxicity on glioma cells (Additional file 1: Fig. S5), we have noticed that YC-1 downregulates the expression of LAMC1 while inhibiting HIF-1α expression at the same time (Fig. 9C, D, Additional file 1: Fig. S4B). Bioinformatics predicted four binding sites for HIF-1α protein on the human *LAMC1* gene promoter (Fig. 9E). The results of promoter luciferase assay showed that HIF-1α directly regulated activity of the *LAMC1* promoter (Fig. 9F). It was noteworthy that we observed strong luciferase activity in cells co-transfected with *LAMC1* Pro-luc and HIF-1α-NC plasmids, suggesting other regulators of *LAMC1* activation in addition to HIF-1α (Fig. 9F).

## Discussion

We comprehensively analyzed the expression and prognostic value of the *LAMC1* gene across pan-cancers. In TCGA database, *LAMC1* was highly expressed in most tumors compared with normal tissues. Kaplan–Meier survival and Cox analyses indicated that high expression of *LAMC1* was associated with a poor prognosis of multiple solid tumor types. Consistent with this result, clinical correlation analysis showed that high expression of *LAMC1* was positively correlated to the stage of most TCGA cancers. Previous reports have suggested that high expression of *LAMC1* is associated with a poor prognosis of certain tumors, such as esophageal squamous cell carcinoma [19], endometrial cancer [8], gastric cancer [20], KIRP [21] and hepatocellular carcinoma [22]. Similar to the results reported above, our current pan-cancer analysis suggested that *LAMC1* expression had a certain clinical relevance in most TCGA tumors, providing global guidance to predict *LAMC1* expression in tumor tissue for clinical prognostic judgment.

An early study of *LAMC1* expression in glioma indicated its prognostic value using the log-rank test and Cox analysis [23]. In the current study, we explored the mechanistic role of *LAMC1* in glioma. The results from TCGA and CGGA databases showed that *LAMC1* expression in gliomas was significantly higher than that in normal brain tissue, and high expression of *LAMC1* was an independent indicator of prognosis. Using a glioma tissue microarray, LAMC1 protein was again found to be highly expressed and positively associated with the pathological grade of glioma. Most importantly, a high level of LAMC1 indicated a poor outcome and shorter OS and DFS. Therefore, on the basis of the bioinformatics analysis of multiple public databases combined with the clinical data validation, *LAMC1* may be a prognostic biomarker of gliomas.

To further reveal the function of the change in *LAMC1* expression during the development and pathological progression of glioma, we used lentiviral vectors to establish cell models with reduced or enhanced *LAMC1* expression. The results of functional experiments in vitro showed significant decreases in cell proliferation, clonogenesis, migration, and invasion after knocking down *LAMC1*, while overexpression of *LAMC1* led to the opposite effects. Some studies have reported on the role of LAMC1 in tumor biology, suggesting that high expression of LAMC1 promotes the proliferation, invasion, and metastasis of tumor cells [19, 20, 22, 24, 25]. Our current results support the above reports. Because of the low tumor formation rate of glioma cells in animals, we were unable to validate in vivo tumor formation and metastasis abilities (data not shown). However, the in vitro results suggest that *LAMC1* plays a crucial role in the biological processes of glioma cells.

Previous studies have suggested that non-coding RNAs play an important role in the regulation of *LAMC1* expression [26–28]. We further explored the mechanisms mediating *LAMC1* expression in gliomas. A hypoxic microenvironment is a major feature of the tumor niche and drives the invasiveness of most solid tumors. And HIF-1α playing a major role in the tumor hypoxic microenvironment [29, 30]. Most importantly, HIF-1α participates in the regulation of tumor biological behavior by reshaping the extracellular matrix in the tumor microenvironment [31]. As an ECM component, the role of *LAMC1* in the tumor hypoxia microenvironment and ECM remodeling is worth further study.

In this study, *HIF-1α* expression was upregulated in most TCGA cancers specifical the solid cancer types. When focusing on gliomas, we found that the high expression of *HIF-1α* accompanied by poor prognosis, higher clinical grade, as well as the status of 1p19q codeletion and IDH mutation; These findings are consistent with previous reports regarding high HIF-1α levels and poor outcome in gliomas [32, 33]. We also found that *HIF-1α* had a positive correlation to *LAMC1* expression in glioma, which led to analyzing the mechanism of HIF-1α-mediated LAMC1 expression. Considering that HIF-1α mainly regulates the expression of downstream genes with hypoxic reaction elements [34], we analyzed the promoter sequence of the *LAMC1* gene and found four potential hypoxic reaction elements. Through luciferase reporter assays of the promoter, we confirmed that HIF-1α directly regulated *LAMC1* promoter activity. However, expression of NC plasmids also enhanced *LAMC1* promoter activity, suggesting that factors other than HIF-1α are involved in regulation of *LAMC1* expression.

Subsequently, glioma cell lines were cultured in a hypoxic environment. After 12 h of hypoxia, an increase in HIF-1α was accompanied by a peak in LAMC1 expression. Moreover, YC-1 inhibited expression of both HIF-1α and LAMC1. More importantly, this was accompanied by decreases in the proliferation and migration of glioma cells. Therefore, the increase in LAMC1 expression in glioma was at least partly mediated directly through HIF-1α. Hence, targeted treatment of the HIF-1/LAMC1 signaling axis may be a novel therapeutic strategy for gliomas. Although an early report mentioned a relationship between LAMC1 and hypoxia [35], our current study clarified the regulatory relationship between HIF-1α and LAMC1 expression, which expands our understanding of the molecular mechanism of ECM remodeling in the hypoxic microenvironment of malignant tumors.

As a major factor involved in tumorigenesis, progression, and the tumor microenvironment, the ECM has become a therapeutic target and potential prognostic marker for gliomas [36]. By considering LAMC1 as target, Kulkarni et al. found that LAMC1 and laminin family members are critical for H-1 parvovirus cell attachment and entry, and can be used as biomarkers to facilitate identifying sensitivity to H-1 parvovirus oncolytic therapy [37]. Therefore, with the development of technology and the improvement in clinical diagnosis and treatment methods, LAMC1 is expected to provide new treatment strategies for malignant tumor patients.

It is worth noting that we have previously reported that *LAMC1* is highly expressed in both KIRP and KIRC, but its prognostic significance is opposite, which may be closely related to its differential immune microenvironment [21]. However, this study found that the expression level of *HIF-1α* in KIRP is not high, indicating that the HIF-1α/LAMC1 signaling axis may not play a role in KIRP, and the reasons are unknown. Meanwhile, as a common sense, it is a hypoxic microenvironment in bone marrow tissue. However, the level of LAMC1 in bone marrow tissue is also not high. As we mentioned the results of *LMAC1* promoter activity experiments, we speculate that there may other yet unknown except HIF-1α regulated LAMC1 expression mechanisms in bone marrow and some solid tumors. The above special features need to be further explored.

## Conclusions

This study demonstrates the biological function, clinicopathological correlation, and clinical prognostic value of *LAMC1* gene expression in pan-cancers, and conducted experimental verification based on glioma. Our investigation revealed that high *LAMC1* expression in some TCGA cancers including glioma indicated a poor prognosis. More importantly, we confirmed the existence of a HIF-1α/LAMC1 axis in the glioma hypoxic microenvironment, which may be a major mechanism regulating its malignant phenotype. Therefore, therapeutic strategies based on inhibition of the HIF-1α/LAMC1 axis to disrupt the tumor hypoxic microenvironment may be a promising approach for glioma treatment.

### Supplementary Information

**Additional file 1: Figure S1.** Screening and targeted intervention of glioma cell lines with differential expression of *LAMC1*. **A** LAMC1 protein expression in glioma cell lines detected by western blotting, *P < 0.05, **P < 0.01. **B** Observation of the lentivirus infection effects in Hs683 cells under microscopes. **C** Western blot was used to detect the inhibitory effects of different RNAi targets on LAMC1 expression, ***P < 0.001 *vs* NC. **D** Observation of the lentivirus infection effects in U251 cells under microscopes. **E** Western blot was used to detect the LAMC1 expression in U251 cells post viral infection, ***P < 0.001. **Figure S2.** Correlation analysis of *LAMC1* expression and clinicopathological features. **A** Age. **B** Stage. **Figure S3.** Immunohistochemical detection of HIF-1α protein expression in clinical gliomas and control tissues. Total 15 sections of clinical samples (normal, n = 3; glioma G1, n = 3; G2, n = 3; G3, n = 3 and G4, n = 3) from Shanghai Outdo Biotech were used for immunohistochemical staining of HIF-1α. Mouse anti human HIF-1α antibody (Cat: #PTM-5851, PTMBIO, Hangzhou, China) and PV-8000 staining kit (ZSGF-BIO, Beijing) were used. The positive expression signal of HIF-1α protein is located in the nucleus and cytoplasm. Expression levels of HIF-1α was quantified using the average optical density (AOD) values, and each section adopts 2 typical views. ***P < 0.001, ****P < 0.0001. **Figure S4.** Expression and regulation of HIF-1α and LAMC1 protein in gastric cancer and hepatocarcinoma cells. **A** LAMC1 and HIF-1α protein expression in gastric and liver cancer cells under normoxia (N) and hypoxia (H) conditions. **B** HIF-1α inhibitor YC-1 inhibited LAMC1 and HIF-1α protein expression in gastric and liver cancer cells after 12 h of hypoxia. **Figure S5.** CCK-8 experiment was used to detect the toxicity of different concentrations of YC-1 on Hs683 cells. ns, no statistical significance. **Table S1**. Relationship of LAMC1 expression with clinicopathological features in glioma. **Table S2**. Univariate and multivariate analyses of the relationship between LAMC1 expression and overall survival of glioma patients.

## Data Availability

Expression profiles of *LAMC1* and *HIF-1α* data were from GTEx, CCLE, TCGA and CGGA databases. All data for validation experiments in this study were presented in the main text and supporting files. Further details are available from the corresponding author upon reasonable request.

## Abbreviations

ACC: Adrenocortical carcinoma
BLCA: Bladder urothelial carcinoma
BRCA: Breast invasive carcinoma
CCK-8: Cell counting kit-8
CESC: Cervical squamous cell carcinoma
CGGA: Chinese Glioma Genome Atlas
CHOL: Cholangiocarcinoma
COAD: Colon adenocarcinoma
DFI: Disease-free interval
DLBC: Diffuse large B-cell lymphoma of lymphoid tumors
DSS: Disease-specific survival
ESCA: Esophageal carcinoma
GBM: Glioblastoma multiforme
HIF-1α: Hypoxia inducible factor-1α
LGG: Lower grade glioma
HNSC: Head and neck squamous cell carcinoma
KICH: Kidney chromophobe
KIRC: Kidney renal clear cell carcinoma
KIRP: Kidney renal papillary cell carcinoma
LAMC1: Laminin subunit gamma-1
LAML: Acute myeloid leukemia
LIHC: Liver hepatocellular carcinoma
LUAD: Lung adenocarcinoma
LUSC: Lung squamous cell carcinoma
MESO: Mesothelioma
OS: Overall survival
OV: Ovarian serous cystadenocarcinoma
PAAD: Pancreatic adenocarcinoma
PCPG: Pheochromocytoma and paraganglioma
PFI: Progression-free interval
PRAD: Prostate adenocarcinoma
READ: Rectum adenocarcinoma
SARC: Sarcoma
SKCM: Skin cutaneous melanoma
STAD: Stomach adenocarcinoma
TCGA: The Cancer Genome Atlas
TGCT: Testicular germ cell tumors
THCA: Thyroid carcinoma
THYM: Thymoma
UCEC: Uterine corpus endometrial carcinoma
UCS: Uterine carcinosarcoma
UVM: Uveal melanoma
