# Supplementary material for: HIF-1α-mediated LAMC1 overexpression is an unfavorable predictor of prognosis for glioma patients: evidence from pan-cancer analysis and validation experiments
Source: J Transl Med. 2024 Apr 27;22:391. doi: 10.1186/s12967-024-05218-3 (PMC11056071; doi:10.1186/s12967-024-05218-3)
Supplement: Supplementary file 1 — Additional file 1: Figure S1. Screening and targeted intervention of glioma cell lines with differential expression of LAMC1. A LAMC1 protein expression in glioma cell lines detected by western blotting, *P < 0.05, **P < 0.01. B Observation of the lentivirus infection effects in Hs683 cells under microscopes. C Western blot was used to detect the inhibitory effects of different RNAi targets on LAMC1 expression, ***P < 0.001 vs NC. D Observation of the lentivirus infection effects in U251 cells under microscopes. E Western blot was used to detect the LAMC1 expression in U251 cells post viral infection, ***P < 0.001. Figure S2. Correlation analysis of LAMC1 expression and clinicopathological features. A Age. B Stage. Figure S3. Immunohistochemical detection of HIF-1α protein expression in clinical gliomas and control tissues. Total 15 sections of clinical samples (normal, n = 3; glioma G1, n = 3; G2, n = 3; G3, n = 3 and G4, n = 3) from Shanghai Outdo Biotech were used for immunohistochemical staining of HIF-1α. Mouse anti human HIF-1α antibody (Cat: #PTM-5851, PTMBIO, Hangzhou, China) and PV-8000 staining kit (ZSGF-BIO, Beijing) were used. The positive expression signal of HIF-1α protein is located in the nucleus and cytoplasm. Expression levels of HIF-1α was quantified using the average optical density (AOD) values, and each section adopts 2 typical views. ***P < 0.001, ****P < 0.0001. Figure S4. Expression and regulation of HIF-1α and LAMC1 protein in gastric cancer and hepatocarcinoma cells. A LAMC1 and HIF-1α protein expression in gastric and liver cancer cells under normoxia (N) and hypoxia (H) conditions. B HIF-1α inhibitor YC-1 inhibited LAMC1 and HIF-1α protein expression in gastric and liver cancer cells after 12 h of hypoxia. Figure S5. CCK-8 experiment was used to detect the toxicity of different concentrations of YC-1 on Hs683 cells. ns, no statistical significance. Table S1. Relationship of LAMC1 expression with clinicopathological features in glioma. Ta [file 12967_2024_5218_MOESM1_ESM.pdf]

Additional file 1

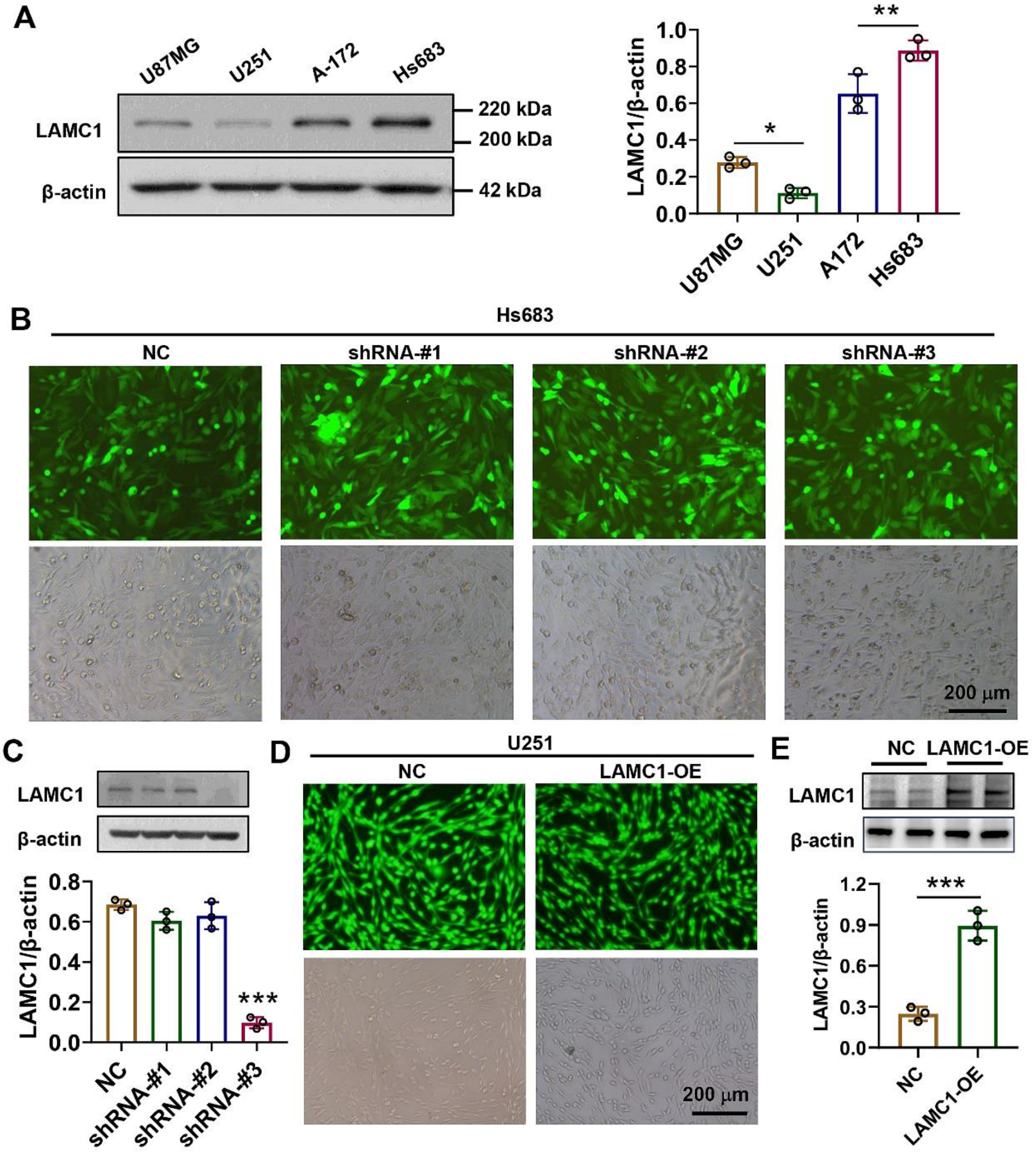

Figure S1. Screening and targeted intervention of glioma cell lines with differential expression of LAMC1. (**A**) LAMC1 protein expression in glioma cell lines detected by western blotting. *$P<0.05$, **$P<0.01$. (**B**) Observation of the lentivirus infection effects in Hs683 cells under microscopes. (**C**) Western blot was used to detect the inhibitory effects of different RNAi targets on LAMC1 expression, ***$P<0.001$ vs NC. (**D**) Observation of the lentivirus infection effects in U251 cells under microscopes. (**E**) Western blot was used to detect the LAMC1 expression in U251 cell line post viral infection, ***$P<0.001$.

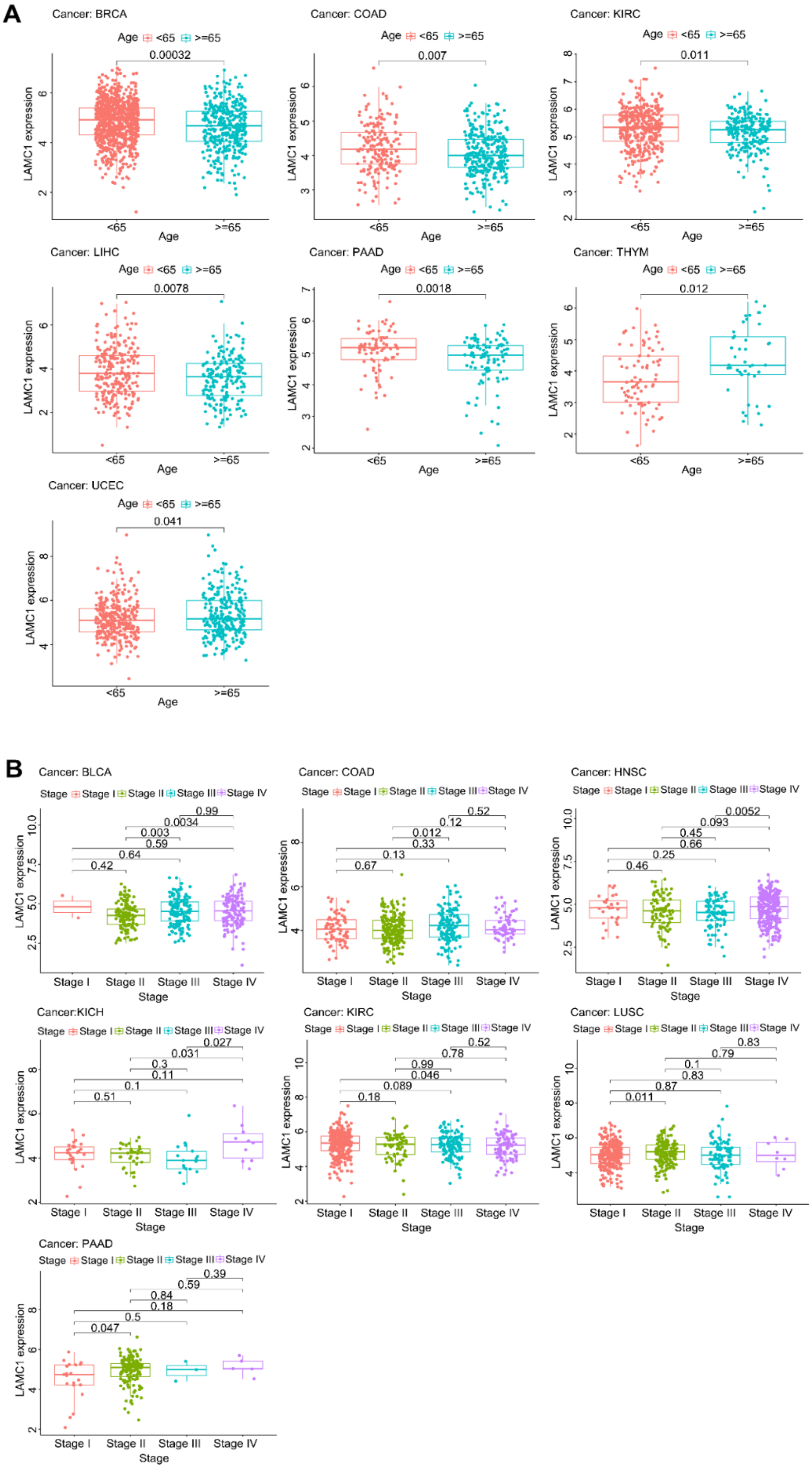

Figure S2. Correlation analysis of *LAMC1* expression and clinicopathological features. (**A**) Age. (**B**) Stage.

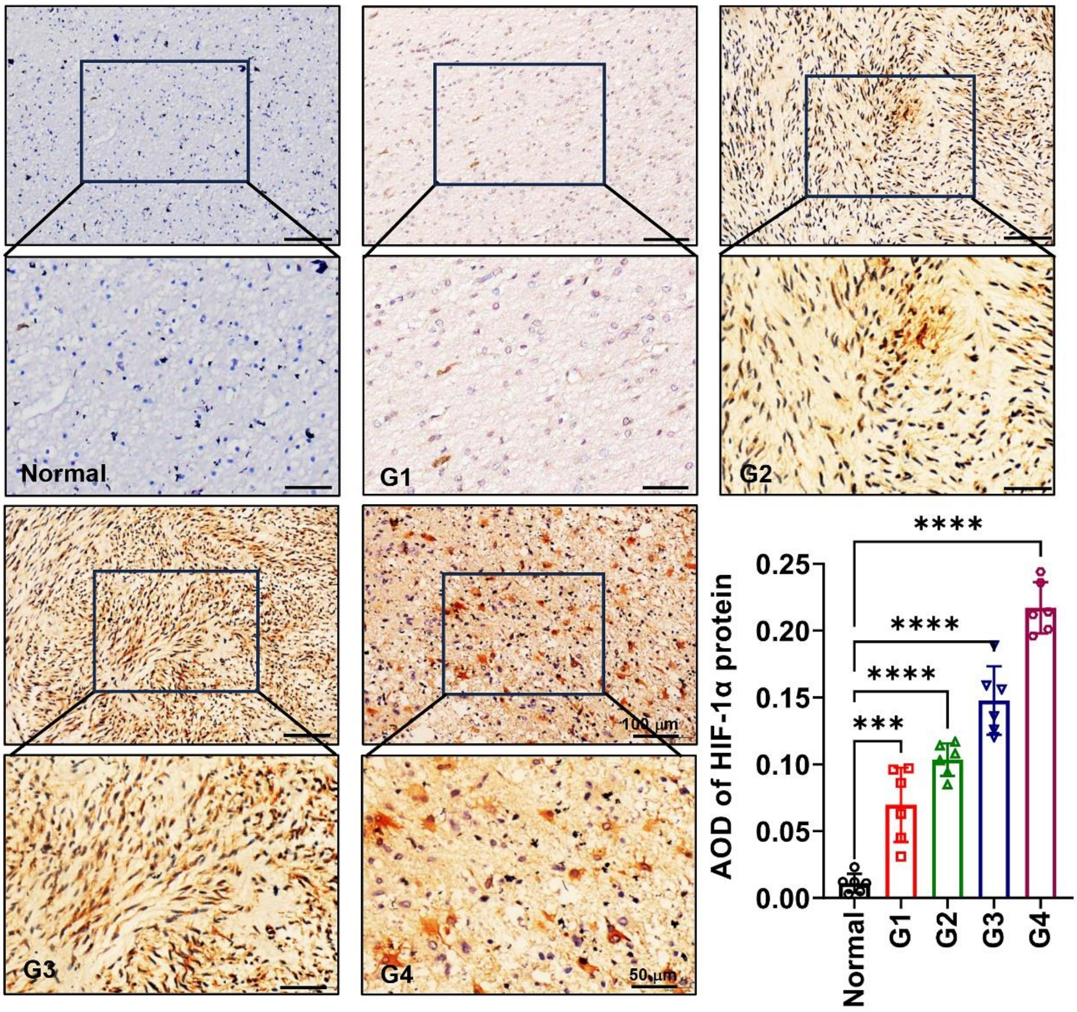

Figure S3. Immunohistochemical detection of HIF-1α protein expression in clinical gliomas and control tissues. Total 15 sections of clinical samples (normal, n=3; glioma G1, n=3; glioma G2, n=3; glioma G3, n=3 and glioma G4, n=3) from Shanghai Outdo Biotech were used for immunohistochemical staining of HIF-1α. Mouse anti human HIF-1α antibody (Cat: #PTM-5851, PTMBIO, Hangzhou, China) and PV-8000 staining kit (ZSGF-BIO, Beijing) were used. The positive expression signal of HIF-1α protein is located in the nucleus and cytoplasm. Expression levels of HIF-1α was quantified using the average optical density (AOD) values, and each section adopts 2 typical views. Bars = 100 μm or 50 μm. ***P<0.01, ****P<0.0001.

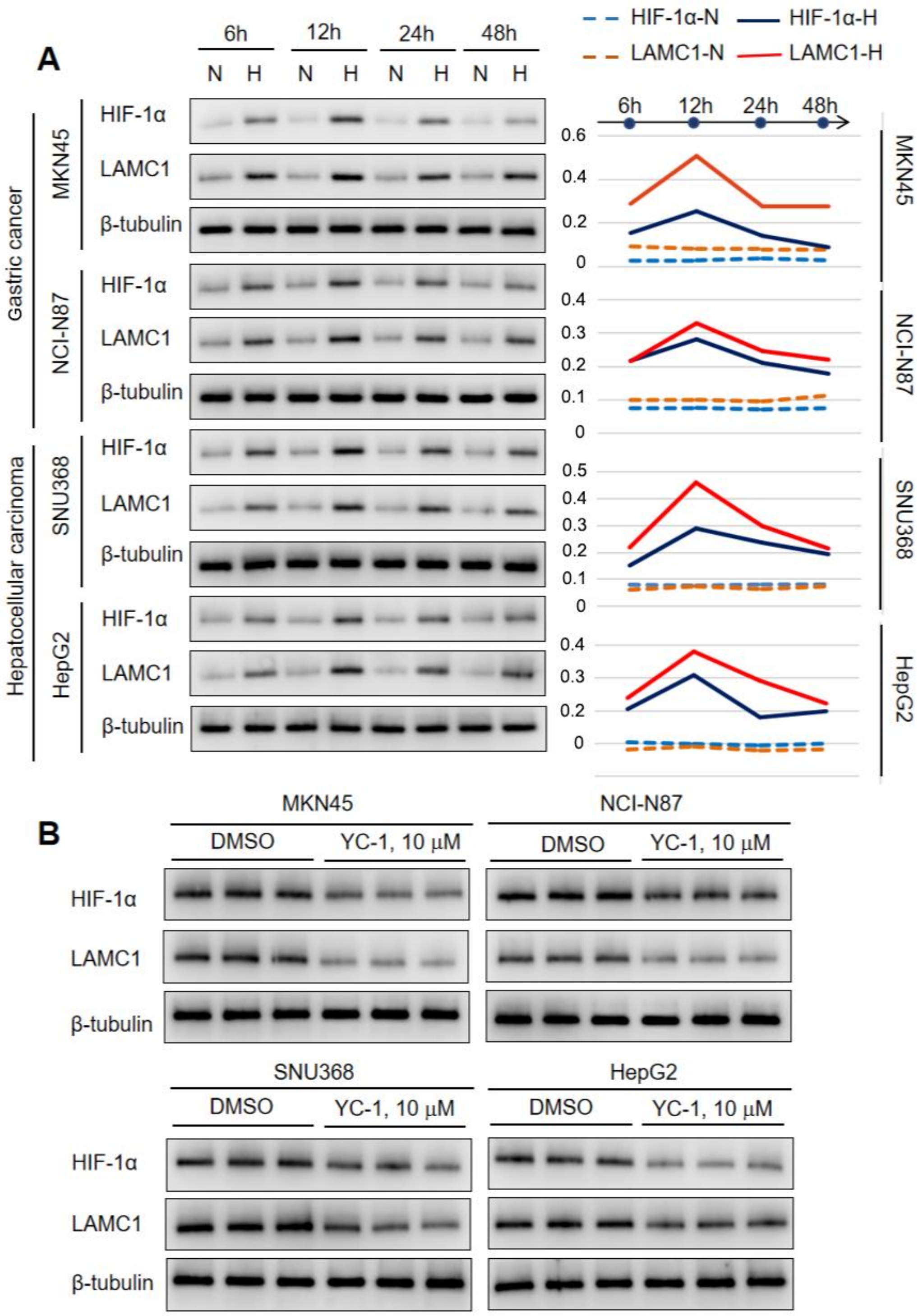

Figure S4. Expression and regulation of HIF-1α and LAMC1 in gastric cancer and liver cancer cells. **(A)** LAMC1 and HIF-1α protein expression in gastric and liver cancer cells under normoxia (N) and hypoxia (H) conditions. **(B)** HIF-1α inhibitor YC-1 inhibited LAMC1 and HIF-1α protein expression in gastric and liver cancer cells after 12 h of hypoxia.

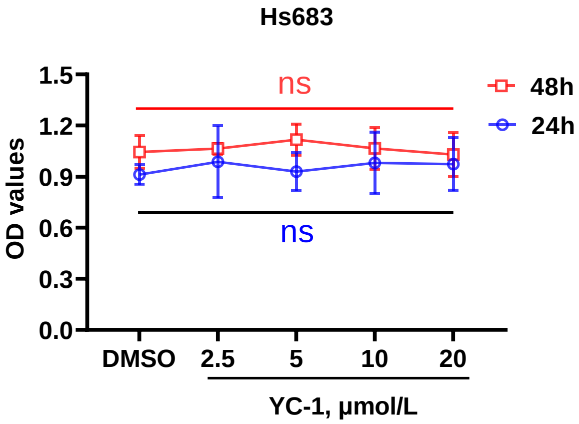

Figure S5. The effect of different concentrations of YC-1 on the viability of Hs683 cells detected by CCK-8 assay. Ns, none significance.

Table S1.

The relation of LAMC1 expression to the clinicopathological features in glioma.

Items		High LAMC1 expression(n=120)	Low LAMC1 expression(n=30)	χ2	P-value
Gender	female	43	10	0.066	0.798
	male	77	20		
Age(years)	≤39	44	14	1.012	0.314
	>39	76	16		
Grade	I-II	68	24	5.510	0.019
	III-IV	52	6		
Postoperative recurrence	No	46	21	9.737	0.002
	Yes	74	9		

Table S2.

Univariate and multivariate analysis of the relationship between LAMC1 expression and overall survival in glioma patients.

Risk factor	Univariate analysis			Multivariate analysis		
	HR	95% CI	P-value	HR	95% CI	P-value
Gender(M/F)	1.559	0.859-2.828	0.144	1.905	1.024-3.545	0.042
Age(>39/ ≤39, years)	3.907	1.908-8.000	0.000	3.964	1.890-8.314	0.000
Grade (I-II/III-IV)	17.821	8.327-38.139	0.000	18.057	8.240-39.573	0.000
Recurrence (Yes/No)	1.460	0.841-2.537	0.179	1.020	0.570-1.824	0.947
LAMC1(High/Low)	2.292	0.980-5.356	0.056	1.138	0.477-2.713	0.770

HR: hazard ratio, CI: confidence interval
